# Supplementary figures and images for: Layered Extraction and Adsorption Performance of Extracellular Polymeric Substances from Activated Sludge in the Enhanced Biological Phosphorus Removal Process
Source: Molecules. 2019 Sep 16;24(18):3358. doi: 10.3390/molecules24183358 (PMC6767238; doi:10.3390/molecules24183358)

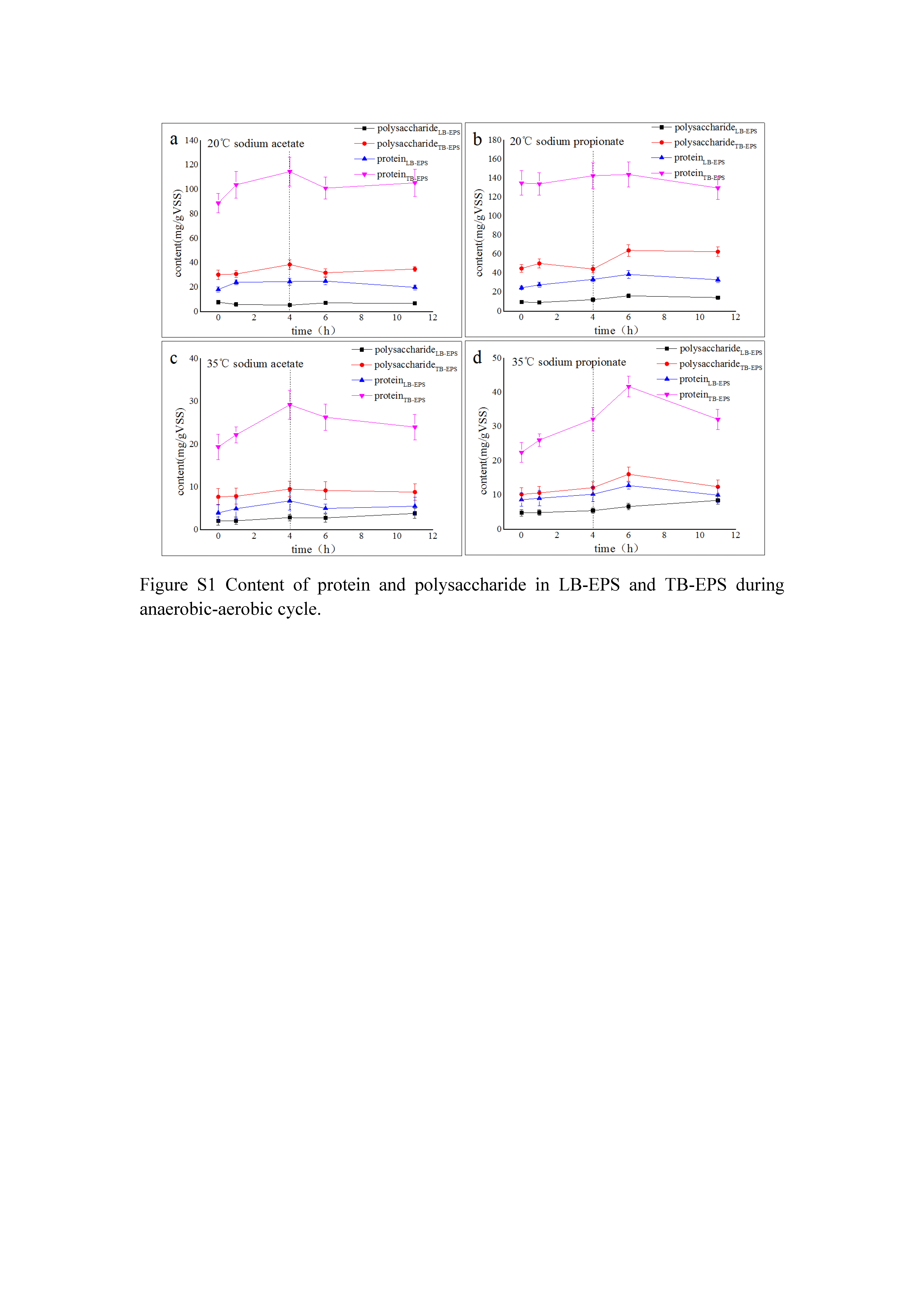

Supplement: Supplementary file 1 [file molecules-24-03358-s001.zip › Figure S1.tiff]

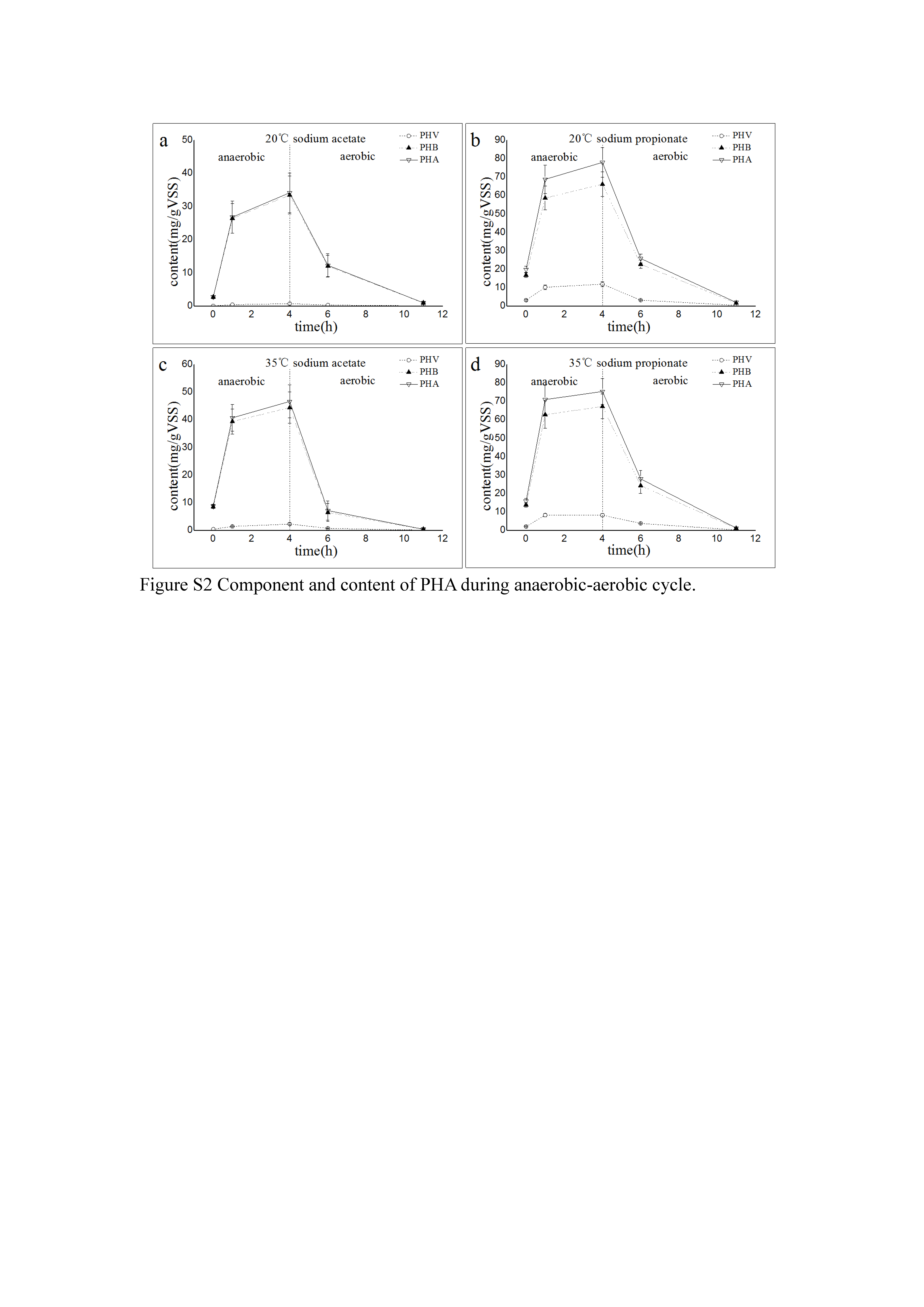

Supplement: Supplementary file 1 [file molecules-24-03358-s001.zip › Figure S2.tif]

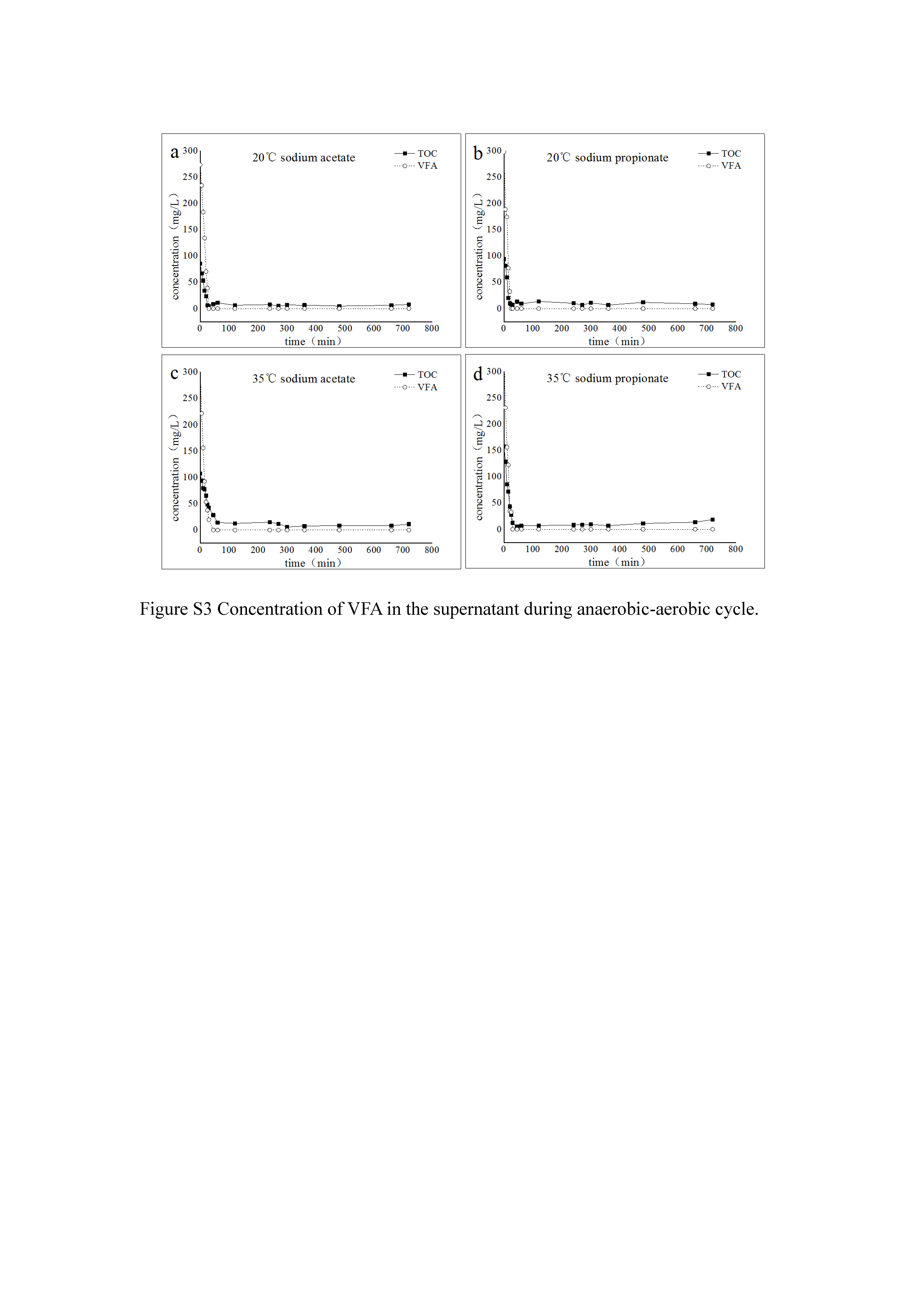

Supplement: Supplementary file 1 [file molecules-24-03358-s001.zip › Figure S3.tif]
