# Supplementary material for: Layered Extraction and Adsorption Performance of Extracellular Polymeric Substances from Activated Sludge in the Enhanced Biological Phosphorus Removal Process
Source: Molecules. 2019 Sep 16;24(18):3358. doi: 10.3390/molecules24183358 (PMC6767238; doi:10.3390/molecules24183358)
Supplement: Supplementary file 1 [file molecules-24-03358-s001.zip › Table S1.doc]

Table S1 The components of synthetic wastewater

| components | concentration（g/L） | components of microelement | concentration（mg/L） |
| --- | --- | --- | --- |
| C_2_H_3_NaO_2_·3H_2_O | 1.2300 | FeSO_4_·7H_2_O | 4.1700 |
| C_3_H_5_NaO_2_ | 1.3800 | MnSO_4_·H_2_O | 1.1300 |
| NH_4_Cl | 0.1433 | ZnSO_4_·7H_2_O | 0.2880 |
| KH_2_PO_4_ | 0.1650 | CoCl_2_·6H_2_O | 0.0840 |
| MgSO_4_·H_2_O | 0.0200 | NaMoO_4_·2H_2_O | 0.0390 |
| CaCl_2_ | 0.0068 | CuSO_4_·5H_2_O | 0.0180 |
| FeSO_4_·7H_2_O | 0.0043 | KI | 0.1080 |
|  |  | H_3_BO_3_ | 0.0900 |
